# N-terminal domain replacement changes an archaeal monoacylglycerol lipase into a triacylglycerol lipase

**DOI:** 10.1186/s13068-019-1452-5

**Published:** 2019-05-06

**Authors:** Surabhi Soni, Sneha S. Sathe, Rutuja R. Sheth, Prince Tiwari, Rajesh-Kumar N. Vadgama, Annamma Anil Odaneth, Arvind M. Lali, Sanjeev K. Chandrayan

**Affiliations:** 1 DBT Centre for Energy Biosciences, Institute of Chemical Technology, Nathalal Parekh Marg, Matunga East, Mumbai, Maharashtra 400019 India; 2 Department of Chemical Engineering, Institute of Chemical Technology, Nathalal Parekh Marg, Matunga East, Mumbai, Maharashtra 400019 India; 3 IISER Mohali, Knowledge City, Sector 81, Manauli PO, Sahibzada Ajit Singh Nagar, Punjab 140306 India

**Keywords:** *Thermococcus onnurineus (strain NA1)*, *Thermomyces lanuginosus* (TLIP), Lipases, Esterases, TON-LPL, rc-TGL, Lid domain, Castor oil

## Abstract

**Background:**

Lipolytic enzymes of hyperthermophilic archaea generally prefer small carbon chain fatty acid esters (C_2_–C_12_) and are categorized as esterases. However, a few have shown activity with long-chain fatty acid esters, but none of them have been classified as a true lipase except a lipolytic enzyme AFL from *Archaeglobus fulgidus*. Thus, our main objective is to engineer an archaeal esterase into a true thermostable lipase for industrial applications. Lipases which hydrolyze long-chain fatty acid esters display an interfacial activation mediated by the lid domain which lies over active site and switches to open conformation at the oil–water interface. Lid domains modulate enzyme activities, substrate specificities, and stabilities which have been shown by protein engineering and mutational analyses. Here, we report engineering of an uncharacterized monoacylglycerol lipase (TON-LPL) from an archaeon *Thermococcus onnurineus (strain NA1)* into a triacylglycerol lipase (rc-TGL) by replacing its 61 N-terminus amino acid residues with 118 residues carrying lid domain of a thermophilic fungal lipase—*Thermomyces lanuginosus* (TLIP).

**Results:**

TON-LPL and rc-TGL were cloned and overexpressed in *E. coli,* and the proteins were purified by Ni–NTA affinity chromatography for biochemical studies. Both enzymes were capable of hydrolyzing various monoglycerides and shared the same optimum pH of 7.0. However, rc-TGL showed a significant decrease of 10 °C in its optimum temperature (T_opt_). The far UV–CD spectrums were consistent with a well-folded α/β-hydrolase fold for both proteins, but gel filtration chromatography revealed a change in quaternary structure from trimer (TON-LPL) to monomer (rc-TGL). Seemingly, the difference in the oligomeric state of rc-TGL may be linked to a decrease in temperature optimum. Nonetheless, rc-TGL hydrolyzed triglycerides and castor oil, while TON-LPL was not active with these substrates.

**Conclusions:**

Here, we have confirmed the predicted esterase activity of TON-LPL and also performed the lid engineering on TON-LPL which effectively expanded its substrate specificity from monoglycerides to triglycerides. This approach provides a way to engineer other hyperthermophilic esterases into industrially suitable lipases by employing N-terminal domain replacement. The immobilized preparation of rc-TGL has shown significant activity with castor oil and has a potential application in castor oil biorefinery to obtain value-added chemicals.

**Electronic supplementary material:**

The online version of this article (10.1186/s13068-019-1452-5) contains supplementary material, which is available to authorized users.

## Background

Lipolytic enzymes catalyze many reactions in aqueous and organic solvents like hydrolysis, transesterification, alcoholysis, aminolysis, acidolysis, and esterification. These unique features put them into a group of extensively used biocatalysts and are used in industries like detergent, food and beverage, pharmaceutical, and biodiesel [1–6]. These enzymes belong to EC 3.1.1.X and are a hydrolase acting on ester bonds of a variety of water-soluble and insoluble esters. At present, there are more than one hundred subclasses in EC 3.1.1.X, based on their substrate specificity or selectivity [7, 8]. Among these, EC 3.1.1.3 is known as triacylglycerol lipase which differs from other esterases regarding substrate specificity, hydrophobicity, and solvent stability. Lipases prefer long-chain fatty acid linked esters ≥ C_10_ and invariably display interfacial activation at the oil–water interface while esterases prefer short-chain fatty acid esters and do not show any interfacial activation [1, 8–12]. Nonetheless, both lipases and esterases belong to same α/β-hydrolase superfamily which also includes many serine proteases as well [11, 13–15]. α/β hydrolases are characterized by the presence of a catalytic triad made up of serine, histidine, and aspartate residues together with an oxyanion hole consisting of backbone amides or positively charged residues. In comparison to other members of the α/β-hydrolase superfamily, most lipases but not all have an additional mobile lid domain located over their active site which rearranges at the oil–water interface.

Robert Kourist et al. have created a α/β-hydrolase fold 3DM database (ABHDB) to organize the diversity in structural features, sequence, and biochemical properties of lipases/esterases [16]. This database is very relevant to provide clues for protein engineering experiments. Based on the composition of the catalytic elbow, the ABHDB has grouped the α/β-Hydrolase superfamily into five families. In these families, lid domains are located between catalytic triad residues except family IV where the lid domain is a N-terminal extension and is connected to overall structure through a long loop. Hence, using a family IV lid domain, it would be straightforward to create a chimeric enzyme with minimum disruption of global structure. Based on the above assumptions, we have designed our N-terminal domain replacement to alter substrate specificity of an archaeal esterase.

Lid domains of lipases are amphipathic having both hydrophilic and hydrophobic amino acid residues and show open and closed conformation in organic and aqueous solvents, respectively [17–19]. This structural switch in an organic solvent from closed to open structure is known as interfacial activation which is a common characteristic of most lipases studied so far. Also, the lid domain has been reported to affect both stability and catalytic properties of the lipase [19]. Therefore, many protein designing experiments have been described in the literature to alter the catalytic feature of lipases such as engineering a disulfide bond in the lid hinge region of *Rhizopus chinensis* lipase showed an increase in thermostability and changed acyl chain length specificity [20]. In another example, increasing the hydrophobicity in the lid domain of *Bacillus thermocatenulatus lipase 2* (BTL2) has shown enhancement in esterification activity for biodiesel production [21].

Lipolytic enzymes are abundant in all three domains of life: eukaryotes, bacteria, and archaea. However, the enzymes studied from hyperthermophilic archaea are mainly esterases having substrate preference towards esters of small-chain fatty acid (C_2_–C_8_). For example, esterase from *Pyrococcus furiosus* [22, 23], *Metallosphera sedula DSM5348* [24]*, Pyrobaculum caldifontis VA1* [25]*, Aeropyrum pernix K1* [26]*, Sulfolobus solfataricus* [27, 28]*, Thermococcus kodakarensis KOD1* [29], *Archaeglobus fulgidus DSM4304* [30, 31], *Pyrobaculum* sp. *1860* [32], and *Sulfolobus acidophilus DSM10332* [33]. Among these, only a lipolytic enzyme AFL from *Archaeoglobus fulgidus* DSM4304 has been categorized as a true lipase based on the presence of a unique C-terminal domain having a small lid domain [30, 34]. Nevertheless, AFL enzyme has been reported to display higher activity to pNP-acetate in comparison of pNP-palmitate [30, 34]. Herein, we report designing of a novel triacylglycerol lipase by replacing the N-terminal domain of an uncharacterized archaeal esterase to a functional lid domain of a lipase (TLIP). The archaeal esterase lysophospholipase (TON-LPL) from a hyperthermophilic archaea *Thermococcus onnurineus (strain NA1)* was overexpressed in *E. coli*. The purified recombinant TON-LPL was active with monoglycerides but did not show activity with triglycerides. Subsequently, we have created a recombinant chimeric enzyme by introducing a lid domain from a well-studied lipase of a thermophilic fungus *Thermomyces lanuginosus* (TLIP). This chimera (rc-TGL) showed activity against triglycerides, castor oil, and olive oil. To the best of our knowledge, this study presents the first example of engineering an archaeal esterase into triacylglycerol lipase.

## Results and discussions

### Designing of a recombinant chimera triacylglycerol lipase (rc-TGL)

A predicted lysophospholipase/monoacylglycerol lipase (TON-LPL) from a hyperthermophilic archaeon *Thermococcus onnurineus* (*strain NA1*) was selected for the lid domain engineering/swapping to design a novel lipase. The sequence analyses of TON-LPL did not anticipate a lid domain in the enzyme (data not shown). Hence, we have inserted a lid domain at N-terminus to enable TON-LPL to act like a triacylglycerol lipase. In our case, the donor of the lid domain was a thermophilic fungal lipase *Thermomyces lanuginosus* (TLIP) which belongs to family IV and has an N-terminal lid domain. Our approach was based on the presumption that rc-TGL will keep the same active site residues and its original structural positions like TON-LPL, but it will have an extra lid domain of TLIP in place of its N-terminal domain.

**Fig. 1:**
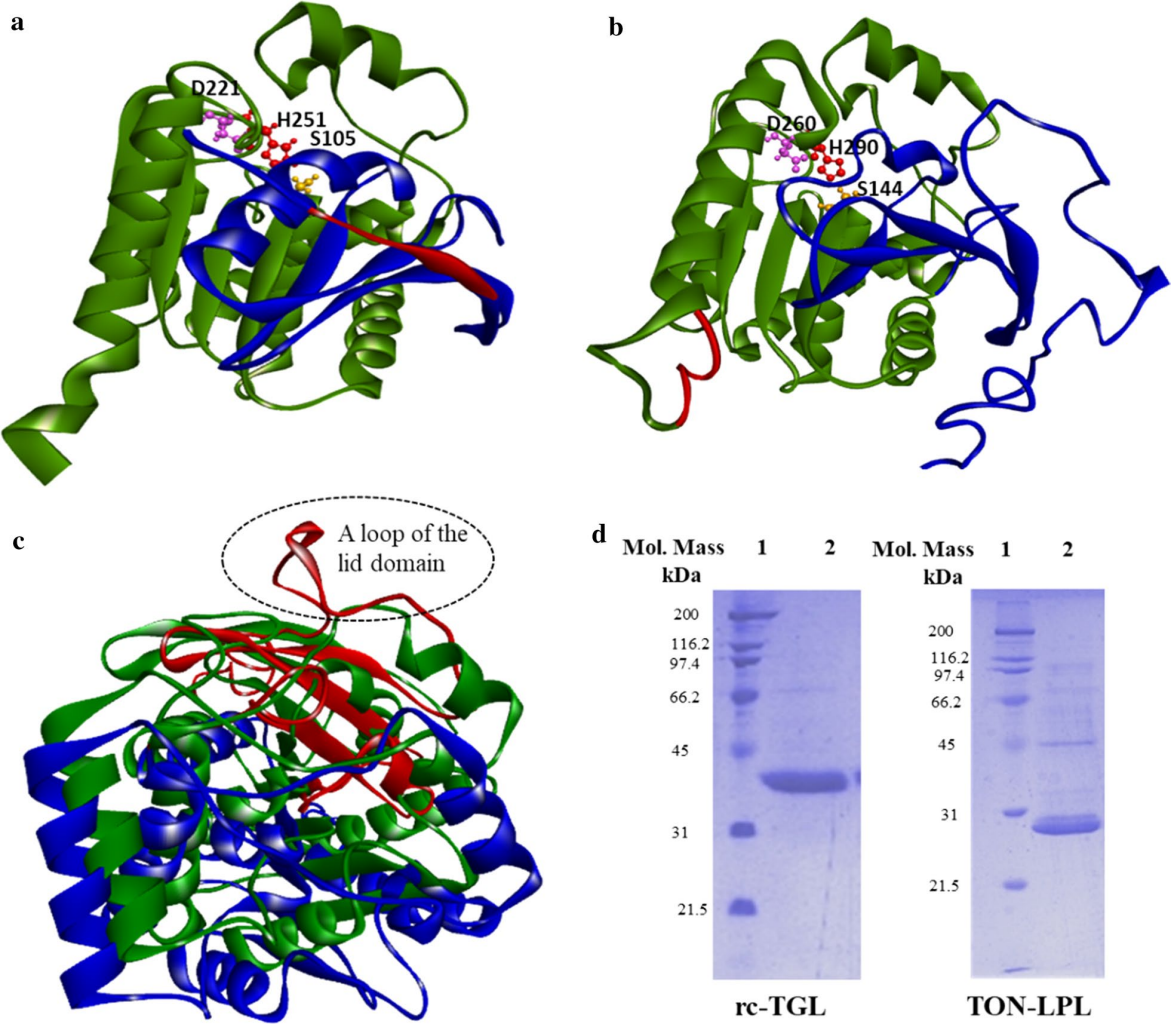
The three-dimensional structural models (**a**–**c**) and SDS-PAGE of Ni–NTA affinity-purified TON-LPL and rc-TGL (**d**). The common structural elements between TON-LPL (**a**) and rc-TGL (**b**) are shown in green, the lid domains used in the N-terminal replacement are shown in blue and the 6×-His tag is in red. The catalytic triad amino acid residues for both models are shown as ball and stick with their amino acid positions (**a**, **b**). The structural superposition of TON-LPL (green) and rc-TGL (blue) is shown in **c**. Here a loop of the lid domain in rc-TGL has been shown in red (dotted black circle). SDS-PAGE of the Ni–NTA affinity-purified TON-LPL and rc-TGL is shown in **d**. Lane 1 is a broad range molecular weight marker and lane 2 is the purified protein and the corresponding purified proteins are named below the SDS-PAGE

TLIP has been well studied and has a three-dimensional structure (PDB: 1DT5), but the structural information of TON-LPL was necessary to design rc-TGL [35, 36]. Therefore, a reliable structural model of TON-LPL was built using a template of a monoglyceride lipase from *Mycobacterium tuberculosis* (PDB ID: 6EIC), which had a reliable C-score of 0.59 having a RMSD (root mean square deviation) of 4.8 ± 3.1 Å. The structural model was generated using I-TASSER (Iterative Threading ASSEmbly Refinement) On-line server [37, 38]. Based on these details, we have defined boundaries for replacing the residues from TON-LPL to create rc-TGL. The structural model of rc-TGL was also built using the same server and same template (selected by algorithm). Both models are shown in Fig. 1a, b. The model of rc-TGL also showed a reliable C-score of 1.65 but has a high value of RMSD of 10.2 ± 4.6 Å. So, RMSD is too high to infer about any role of a particular residue in the overall structure of rc-TGL. Yet, the structural superposition of both models was carried out to assess the gross structural differences between TON-LPL and rc-TGL (Fig. 1c). Based on these, a long loop from the lid domain was noticed into rc-TGL over the active site which was missing in TON-LPL (Fig. 1c). Noticeably, the catalytic residues in the active site pocket of rc-TGL were also perceptively repositioned in comparison of TON-LPL (Fig. 1c). In addition, there were also recognizable subtle changes in the positioning of secondary structural elements between both models. Therefore, we assume the biochemical characteristics of rc-TGL may differ in comparison to TON-LPL.

Recombinant TON-LPL is 281 residues long in which 18 residues are additional and consist of 6×His affinity tag located at N-terminus and a few residues from an expression vector. rc-TGL was constructed by replacing the continuous stretch of 61 residues from N-terminus of TON-LPL to 118 residues of TLIP. Overall, recombinant rc-TGL is made of total 329 residues of which 203 residues are from TON-LPL, 118 residues from TLIP, and eight residues are from expression vector containing a 6xHis tag located at C-terminus. The amino acid sequences of TLIP, TON-LPL, and rc-TGL are depicted in Additional file 1.

### Cloning, expression, and Purification of TON-LPL and rc-TGL

TON-LPL gene was PCR amplified from the genome of *Thermococcus onnurineus* (*strain NA1*) and the PCR fragment was cloned into a pQE30 expression vector at *KpnI* and *HindIII* restriction sites. The obtained recombinant plasmid carrying TON-LPL gene was transformed into XL1 Blue cells for overexpression. Protein expression was achieved by inducing the XL1 Blue by 1 mM IPTG at mid-exponential phase (Additional file 2A). Initial attempts of Ni–NTA affinity chromatography (IMAC) by loading the cell-free extract under native buffer conditions (buffer without 8 M urea) were unsuccessful in yielding purified TON-LPL. However, overexpressed histidine-tagged TON-LPL was present in the cell-free extract (Additional file 2A). Hence, an alternative method was employed where 8 M urea was included into the lysis buffer and the protein was captured onto column in the same urea containing lysis buffer. After binding, the column was washed, and protein was eluted in a urea-free refolding buffer. This process of on-column refolding of TON-LPL resulted into overall protein yield of 2–3 mg/g wet cells. Subsequently, the molecular weight of the affinity-purified TON-LPL was determined by SDS-PAGE (Fig. 1d). Based on SDS-PAGE, the molecular weight was ~ 29–30 kDa which was in agreement with the theoretical average molecular weight of 31.2 kDa.

rc-TGL gene was synthesized from SOE-PCR (splicing overlap extension PCR) and the details have been described in Additional file 3: 3.1, 3.2, and 3.3. After our unsuccessful attempts to clone, express, and purify rc-TGL using pQE30 expression plasmid, we have successfully cloned and overexpressed it into pET23a expression plasmid. The failure to get a significant amount of rc-TGL in pQE30 can be explained by the difference in mechanism of protein folding and the rate of translation in different bacterial hosts under dissimilar promoters [39]. Overexpressed rc-TGL was purified by Ni–NTA affinity chromatography under native buffer condition (in urea-free buffer) unlike to TON-LPL. The purified rc-TGL is shown in Fig. 1d and showed an approximate molecular weight ~ 35–36 kDa, which is consistent with the theoretically calculated molecular weight of 36.2 kDa.

The observed differential performance of the affinity tag in Ni–NTA affinity purification could be explained by an assumption that the location of the 6×-histidine tag in an enzyme influences its accessibility to the Ni–NTA resin [40]. Here, in TON-LPL, the 6×-histidine tag was at N-terminus, whereas, in rc-TGL, it was at C-terminus. Correspondingly, the structural models (Fig. 1a, b) speculate that the C-terminus tag was more exposed in comparison to N-terminus tag of TON-LPL (Fig. 1a, b). Thus, the tag located at C-terminus (rc-TGL) showed better binding to Ni–NTA affinity column in comparison of an N-terminus affinity tag (TON-LPL). Nonetheless, the refolded TON-LPL having an N-terminal tag showed better binding (Additional file 2), which suggest that overexpressed TON-LPL in cell-free extract was folded differently to the refolded TON-LPL.

### Temperature, pH optima, and substrate specificity of TON-LPL and rc-TGL

**Fig. 2:**
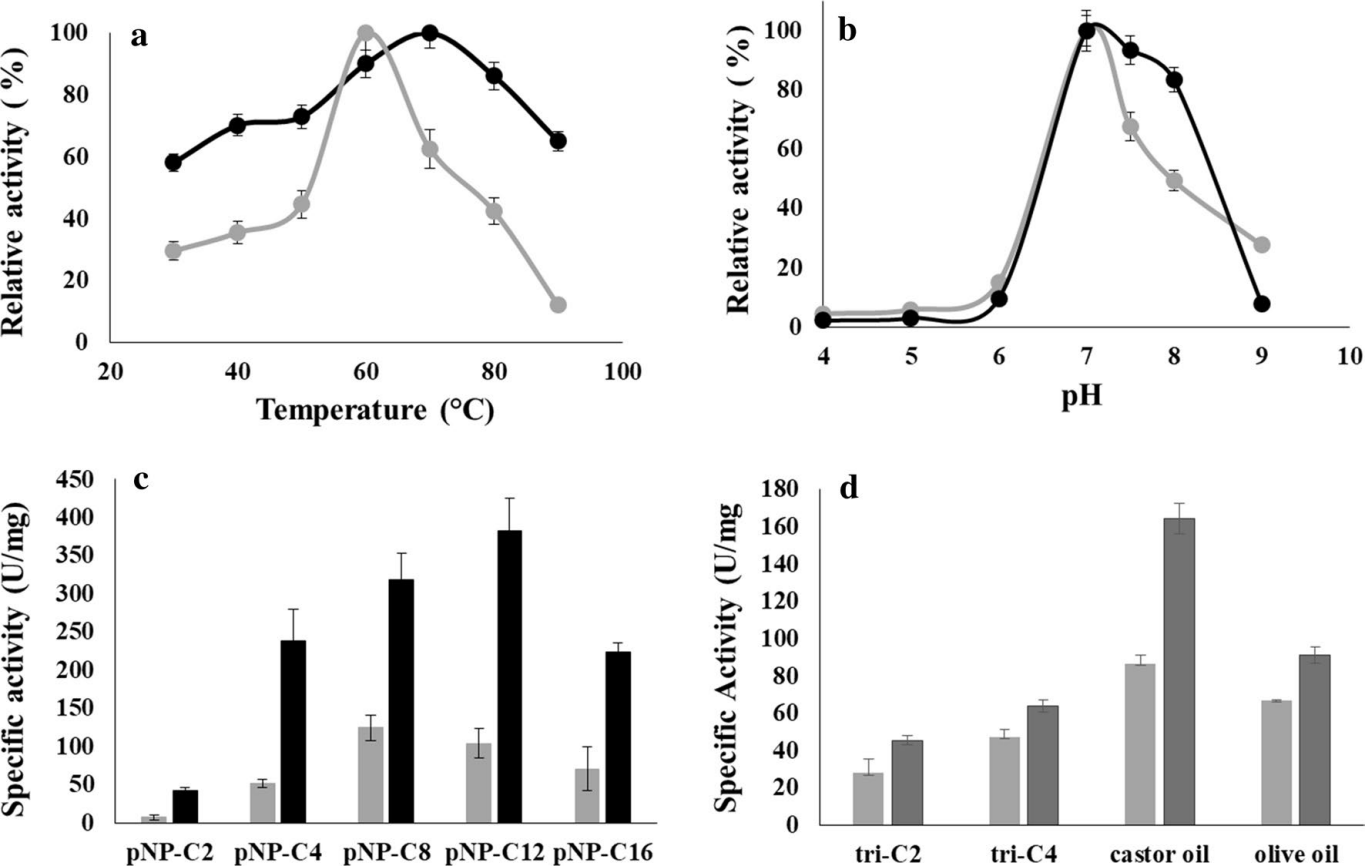
Biochemical characterization of TON-LPL and rc-TGL. Relative activities at different temperatures and pH are shown in **a**, **b** for TON-LPL (black) and rc-TGL (gray), respectively. All reactions were incubated for 5 min and performed in triplicate with pNP-butyrate. For different pH, the incubation temperature for TON-LPL and rc-TGL was 70 °C and 60 °C, respectively. The specific activity with different substrates; pNP C-2 (acetate); pNP C-4 (butyrate); pNP C-8 (octanoate), pNP C-12 (dodecanoate) and pNP-C-16 (palmitate) is shown in **c** for TON-LPL (black) and rc-TGL (gray). All assays with triglycerides were performed in triplicates and the enzymatic activity was determined by the titration method and shown in rc-TGL (gray) and TLIP (light black). The specific activity with different triglyceride substrates; Tri-C2, Tri-C4, castor oil, and olive oil is shown in **d**. All assays were performed in triplicates

pNP-butyrate was used as a substrate to determine temperature and pH optima for purified TON-LPL and rc-TGL. TON-LPL was active at a narrow pH range from 7.0 to 8.0 but rc-TGL had a broad pH range from 7.0 to 9.0 (Fig. 2b). However, the optimum pH values of both enzymes were at pH 7.0. Subsequently, at pH 7.0, the activity was determined in a temperature range of 30-90 °C. TON-LPL showed significant activity up to 90 °C with an optimum at 70 °C (Fig. 2a). In comparison, rc-TGL showed the optimum temperature of 60 °C, and beyond this, it showed a rapid drop in activity. The structural perturbations are expected to alter the protein stability and activity and the likely molecular mechanism explaining a shift in the temperature optimum is discussed in the later section.

The specific activity of both the enzymes was evaluated with different monoglyceride substrates pNP C-2, pNP C-4, pNP C-8, pNP C-12, and pNP C-16. However, the maximum activity of TON-LPL was with pNP-C12, while rc-TGL was most active with pNP-C8 (octanoate). Nevertheless, both enzymes had a significant activity with pNP-C16 esters (Fig. 2c). Notably, as compared to TON-LPL, the specific activity of rc-TGL was 2–3-fold lower on abovementioned substrates. Conclusively, this N-terminal lid replacement has significantly changed overall enzyme activity and stability of TON-LPL. Nevertheless, our N-terminal lid replacement strategy has yielded an active rc-TGL.

Further, we tested the activity against triglyceride substrates—triacetin, tributyrin, castor oil, and olive oil. In these experiments, TLIP was used as a positive control. As expected, TON-LPL was not active with any substrates (data not shown), whereas rc-TGL was active with all substrates and the maximum activity was observed with castor oil (Fig. 2d). However, in comparison to TLIP (positive control), the activity was reduced to approximately 40–50%. The observed catalysis confirms the functionality of the exchanged lid domain to TON-LPL for the creation of rc-TGL. Combined all the above data, we have biochemically characterized TON-LPL as a monoacylglycerol lipase and successfully engineered it into a triacylglycerol lipase (rc-TGL).

### Thermostability of TON-LPL and rc-TGL

**Fig. 3:**
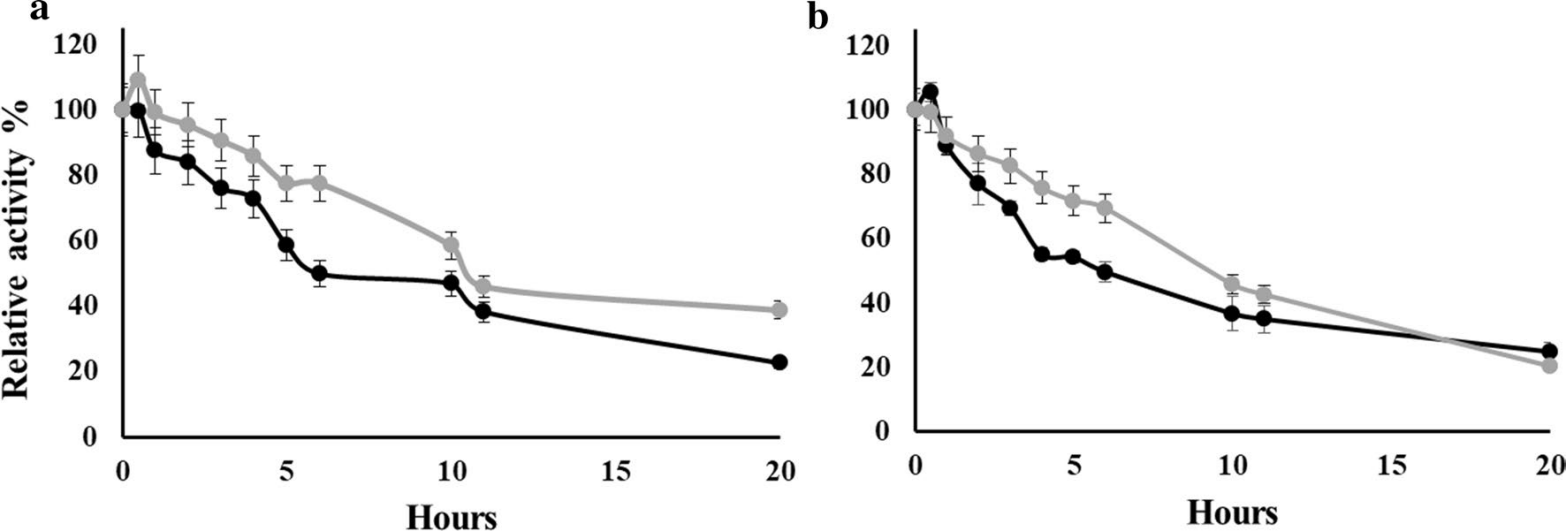
Thermal inactivation kinetics of TON-LPL (black) and rc-TGL (gray). TON-LPL and rc-TGL were incubated at 60 °C and 70 °C in a 100 mM phosphate buffer of pH 7.0 (**a** and **b**, respectively). At different time intervals, the aliquots were taken and followed by an assay with pNP-butyrate as described in method section. All assays were performed in triplicates, and relative activities are shown

As discussed previously, the temperature optimum of rc-TGL and TON-LPL was at 60 °C and 70 °C, respectively. Hence, thermal inactivation profiles were determined at 60 and 70 °C for both proteins (Fig. 3a, b). At 60 °C, both enzymes showed similar profile and had very comparable half-life of approximately 10.0 h. The inactivation profiles for both enzymes at 70 °C were also very alike and both enzymes had an indistinguishable half-life of approximately 7.0 h.

**Fig. 4:**
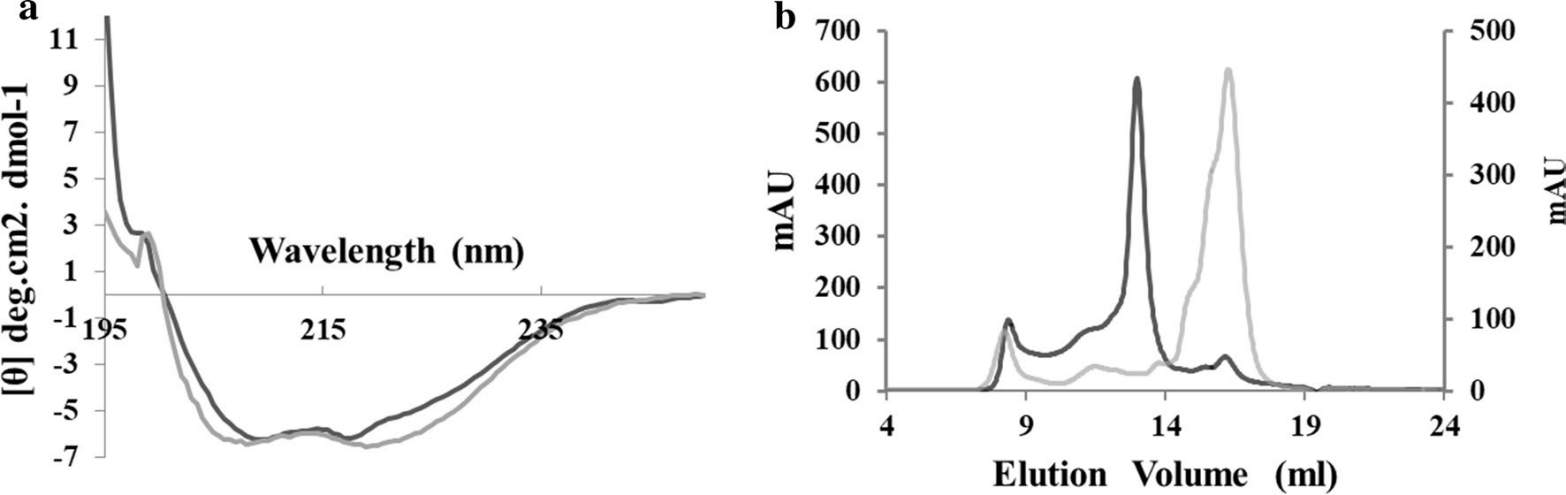
Secondary and quaternary structure analyses. **a** The far UV–CD spectrum of TON-LPL (black) and rc-TGL (gray) in 100 mM sodium-phosphate buffer of pH-7.5. **b** Gel filtration profile of TON-LPL (black) and rc-TGL (gray) obtained from Superdex 200 increase column in a 100 mM Sodium-phosphate buffer of pH-7.5 with 300 mM NaCl

Subsequently, we did far UV–CD to find the pattern of global secondary structure in both active enzymes. Both enzymes displayed a typical peak for α-helix at 208 nm and 222 nm and β-sheet at 218 nm (Fig. 4a). Hence, TON-LPL and rc-TGL were well-folded like a typical α/β hydrolase fold domain. Following this, the quaternary structure was analyzed by gel filtration chromatography, in which TON-LPL showed a molecular weight of ~ 110 kDa corresponding to a trimer or a compact tetramer while rc-TGL showed a molecular weight of ~ 29 kDa corresponding to a compact monomer (Fig. 4b and Additional file 4). The molecular weights and oligomeric states of TON-LPL and rc-TGL were calculated based on a standard calibration curve as shown in Additional file 4. Interestingly, in spite of similar global secondary structure, TON-LPL and rc-TGL were different in their oligomeric state. Oligomerization has been proposed as one of the mechanisms to gain thermostability and which is quite evident in proteins from hyperthermophilic organisms [41–43]. Some of them have also shown loss of thermostability upon disruption of the oligomeric structure. For example, crystal structural and mutational analyses of l-isoaspartyl-*O*-methyltransferase from *Sulfolobus tokodaii* [43] and β-glucosidase from *Pyrococcus furiosus* [44]. Thus, the observed shift of temperature optima of rc-TGL may arise from the change of trimeric TON-LPL into monomeric rc-TGL. Moreover, N-terminal residues of TON-LPL seem to play a crucial role in the formation of the interface of oligomerization in TON-LPL because we have replaced the N-terminus of the same.

Numerous studies have observed a tradeoff between, the protein function and stability during protein evolution, and in our case, we also report that TON-LPL loses its stability to broaden its function in our protein engineering experiments. Likewise, our protein engineering experiments also support the view of stability-function tradeoff in protein evolution [45–47].

### Enzyme immobilization and castor oil hydrolysis by rc-TGL

**Fig. 5:**
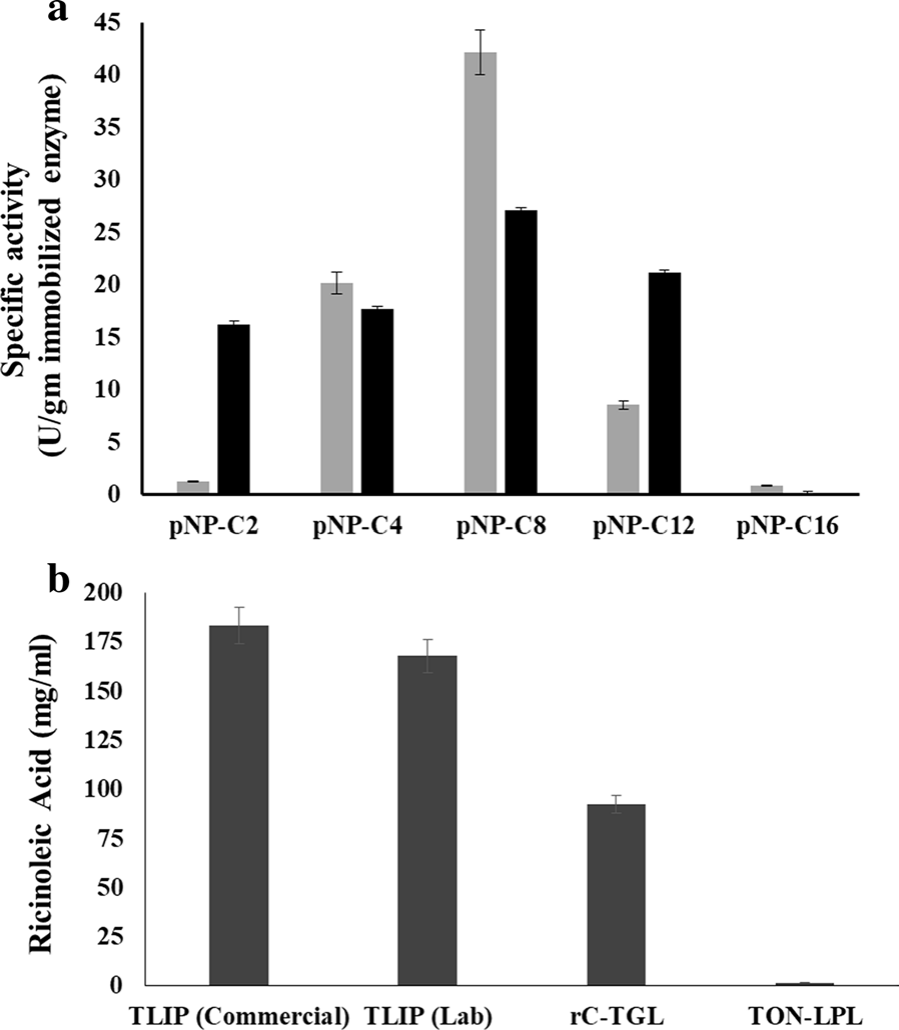
Biochemical characterization of immobilized enzymes. **a** Relative activity of immobilized TON-LPL (black) and rc-TGL (gray) with different substrates pNP C-2 (acetate); pNP C-4 (butyrate); pNP C-8 (octanoate), pNP C-12 (dodecanoate), and pNP-C-16 (palmitate). Here the enzyme units are expressed in per gram of resin having bound enzymes. **b** HPLC-based quantification of ricinoleic acid (mg/ml) produced by hydrolysis of castor oil. The standard curve or ricinoleic acid and HPLC profiles of castor oil hydrolysis are shown in Additional file 6

rc-TGL showed significant activity with castor oil in aqueous buffer so, we also evaluated its potential application in castor oil biorefinery as an immobilized enzyme. Therefore, both enzymes were successfully immobilized by physical adsorption on the Indion^®^ PA 500 resin, a non-ionic, hydrophobic crosslinked polymer within 2 h (Additional file 5A, B). Immobilization of TON-LPL and rc-TGL was monitored by following the pNP-butyrate units at different steps of immobilization process (Additional file 5A, B). TON-LPL showed ~ 20–25% increase in total enzyme units/g of resin, while rc-TGL showed a drop of 10% in total enzyme units/g of resin upon immobilization (Additional file 5). Moreover, immobilized rc-TGL also lost its activity towards long-chain fatty acid ester while TON-LPL did not show any change in its substrate profile after immobilization (Fig. 5a). Probably rc-TGL has acquired some conformational change during the immobilization process which presumably causes enzyme inactivation upon immobilization. Even so, the immobilized rc-TGL was tested for castor oil hydrolysis in organic solvent with two different preparations of immobilized TLIP (lab prepared and commercial) as a positive control. The castor oil hydrolysis was performed in tertiary butanol with a small volume of water and reaction products were analyzed by HPLC for detection of released ricinoleic acid. TON-LPL did not hydrolyze castor oil at all, but it was interesting to see that rc-TGL did hydrolyze and produce ricinoleic acid (Additional file 6a, b). However, the yield of ricinoleic acid for rc-TGL was decreased to ~ 50–55% in comparison of commercial immobilized TLIP (Fig. 5b). We have also used an increased amount of immobilized rc-TGL and found that the product was linearly increasing for rc-TGL but not for TON-LPL (data not shown). Thus, the behavior of immobilized rc-TGL was indicative of a switch in lid conformation from closed to open at the oil–water interface.

## Conclusion

We have chosen a predicted monoglyceride lipase (TON-LPL) from a hyperthermophilic archaeon *Thermococcus onnurineus* (*strain NA1*) and have successfully changed it into a triglyceride lipase through lid engineering approach. As expected, TON-LPL was active with only monoglyceride substrates and displayed the temperature and pH optima of 70 °C and 7.0, respectively. Subsequently, we have replaced the first 61 residues of TON-LPL from N-terminus with 118 residues carrying a functional lid domain from a thermophilic fungal lipase from *Thermomyces lanuginosus* (TLIP). This chimera (rc-TGL) was successfully overexpressed in *E. coli* and purified to homogeneity. Like TON-LPL, rc-TGL was also active with monoglycerides and had similar pH optimum. In contrast, the chimera showed a significant difference in temperature optimum by 10 °C. The change in quaternary structure of rc-TGL from trimer to monomer has been explained as a cause for a shift in the temperature optimum. Nonetheless, rc-TGL acquired a functional lid domain and was active with triglyceride substrates and castor oil as well. Our work proves an approach to engineering an esterase or monoglyceride lipase into triglyceride lipase. The observed activity of immobilized rc-TGL with castor oil shows the potential application of this enzyme in castor oil biorefinery [48, 49].

## Methods

### Gene cloning, splicing overlap extension PCR (SOE-PCR), and cell growth for protein overexpression

The gene encoding TON-LPL (NCBI Reference Sequence ACJ16398) from *Thermococcus onnurineus* NA1 was PCR amplified using Prime STAR HS DNA polymerase premix (DSS Takara Bio India Pvt. Ltd., New Delhi, India).The forward primer-5′-ATTTTA*GGTACC*GTGAGGGTTTACAAAGCCAAGTTCGGTGAGC-3 and reverse primer-5′-TTAATA*AAGCTT*ATTCTCTTTTCTTCCTCCCGCGTG-3′ (restriction sites are written in italics) were used in the PCR. The PCR amplified product were digested by *KpnI* and *HindIII*. Subsequently, it was ligated into the predigested pQE30 vector. The ligation products were transformed into XL1 Blue, and positive colonies were selected for protein overexpression studies. Protein was expressed by growing the cells at 37 °C for 24 h with constant shaking at 200 rpm in 1 L terrific broth autoinduction medium with trace elements (AIM-Terrific Broth, Hi-Media Laboratories Pvt. Ltd., Mumbai, India) or in LB broth (induced by 1 mM IPTG).

The chimera gene encoding rc-TGL was constructed by splicing overlap extension PCR (SOE-PCR) reaction using two gene fragments: one fragment was amplified from *Thermomyces lanuginosus* lipase gene (TLIP), and another fragment was from TON-LPL gene. The forward and reverse primers used are listed in Additional file 3: 3.3. The SOE-PCR amplified product was digested by *NdeI* and *XhoI* and ligated into pET-23a vector having a 6×-His tag at its C-terminus. The ligation products were transformed into XL1 Blue and the positive clones were confirmed by restriction digestion analysis of the isolated plasmid (Additional file 3: 3.2). The pET-23a plasmid carrying rc-TGL gene was transformed into BL21 (DE3) cells for protein expression. Protein was expressed by growing the BL21 (DE3) cells at 37 °C for 24 h in 1 L terrific broth autoinduction medium with trace elements (Hi-Media Laboratories Pvt. Ltd., Mumbai, India).

### Protein purification, quantification, and electrophoresis

Overnight grown cells overexpressing TON-LPL and rc-TGL were lysed in a denaturing lysis buffer (8 M Urea, 1 M NaCl, 100 mM pH 7.5) and native lysis buffer (300 mM NaCl, 5 mM Imidazole, 0.1 mg/ml lysozyme in 100 mM pH 7.5), respectively, by repeated freeze–thaw in liquid nitrogen. The Ni–NTA agarose column (Qiagen, Venlo, Netherlands) was pre-equilibrated with corresponding lysis buffers. In both cases, bound proteins were eluted in a non-denaturing buffer (1 M imidazole, 300 Mm NaCl, and 100 mM phosphate buffer of pH-7.5). Protein estimation in elution fractions was done by Bradford method, and purified enzymes were analyzed by SDS-PAGE to assess the purity and molecular weight according to Laemmli SDS-PAGE. The affinity-purified TON-LPL and rc-TGL was further buffer exchanged in a 3 kDa Microsep™ Advance Centrifugal Devices (Pall Corporation, Pune India) and quantified based on their molar extinction coefficient at 280 nm.

### Enzyme assays

10 mM stock solutions of pNP-acetate, pNP-butyrate pNP-octanoate, pNP-dodecanoate, and pNP-palmitate were prepared in 100% isopropanol and were purchased from Sigma-Aldrich, St Louis, USA. In the assay mixture, 0.25 mM substrate was used with ~ 0.2 µg TON-LPL and rc-TGL in a total reaction volume of 2 ml. The reaction was carried out in a Thermomixer (Eppendorf, Germany) for 15 min and released pNP after hydrolysis was measured at 410 nm in a Shimadzu UV–visible spectrophotometer. One unit of enzyme corresponds to release of 1 µmol pNP/minute/ml, and specific activity corresponds to enzyme units/mg. Measurements at different pH were performed in following buffers: 100 mM citrate buffer for pH 5.0–6.0, 100 mM phosphate buffer for pH 7.0–7.5, and 100 mM tris buffer for pH 8.0–9.0. At pH-7.5, assays were also performed in a temperature range of 30–90 °C to determine the optimum temperature. Enzyme units calculations at different pH were based on corresponding molar extinction coefficient at 410 nm, and those were 1.8, 9.6, 13.3, 17.4, and 18.1 L^−1^ mmol^−1^ cm^−1^ at pH-6.0, pH-7.0, pH-7.5, pH-8.0, and pH-9.0, respectively. All the enzyme assays were performed in triplicates with their corresponding enzyme blank control, and the presented data are the average of those three measurements.

For triglyceride hydrolysis, the enzyme units were calculated by titration of the released fatty acid with 0.5 M NaOH and the assays were performed for TON-LPL (70 °C) and rc-TGL (60 °C) in 100 mM phosphate buffer of pH 7.5. All the enzyme assays were performed in triplicates with their corresponding enzyme blank control. For these assays, the substrates used were triacetin, tributyrin (both procured from Sigma-Aldrich, St Louis, USA), castor oil, and olive oil (from the local market).

The thermal stability of both enzymes was determined by incubating the protein solution in 100 mM pH 7.5 phosphate buffer at their optimum temperatures and at different time points, assays were performed using the protocol as mentioned earlier.

### Gel filtration chromatography

The purified and concentrated TON-LPL (0.5 mg) and rc-TGL (0.5 mg) were loaded on a Superdex 200 increase column (GE Healthcare, USA), pre-equilibrated with running buffer of pH 7.5 (100 mM sodium phosphate, 300 mM NaCl) at a flow rate of 0.5 ml/min on the ÄKTApurifier GE Healthcare. The molecular weights were estimated by running a following gel filtration standards-Ferritin (440 kDa), Conalbumin (75 kDa), Ovalbumin (43 kDa), Carbonic anhydrase (29 kDa), Ribonuclease A (13.7 kDa), and Aprotinin (6.5 kDa), under similar running conditions on the same column and a standard calibration curve of log molecular weight versus Ve/Vo (Ve: elution volume and Vo: void volume) is shown in Additional file 4.

### Far UV–CD spectrum

The elution fractions of gel filtration chromatography were collected and used for far UV–CD spectrum on a CDBIO-KINE MOS-500 spectrometer with acquisition duration of 0.5 s, the bandwidth of 2 nm in a 2 mm cuvette. All spectrum represents the average of 20 scans, and protein concentrations of TON-LPL and rc-TGL were 0.2 and 0.36 mg/ml, respectively. The obtained raw ellipticity (mdeg) was converted into molar residue ellipticity (MRE), where MRE = mdeg * 100 * MRW/1000 * concentration (mg/ml)*path length (in cm) and MRW = molecular weight in Da/number of amino acids.

### Enzyme immobilization

Purified and concentrated TON-LPL and rc-TGL in 100 mM phosphate buffer (pH 7.5) were immobilized onto polystyrene divinyl-benzene beads of Indion PA 500 resin **(**Ion Exchange India Ltd.**)** by physical adsorption at 37 °C with a shaking speed of 180 rpm for 2–3 h. The immobilization was confirmed by running supernatant samples at regular interval on SDS-PAGE as well as pNP units were determined for all samples (Additional file 5). Both immobilized enzymes were also characterized by enzyme assays as described earlier.

### HPLC analysis of castor oil hydrolysis

One-gram castor oil was dissolved in a medium containing 4.95 ml of tertiary butanol solvent and 0.05 ml of water. The reaction was initiated by addition of 200 pNP enzyme units of each immobilized enzyme (TON-LPL, rc-TGL, and TLIP). The reaction mixture was incubated at 50 °C with shaking at 200 rpm for 8 h, and at end 50 µl samples were taken and dissolved into 450 µl tertiary butanol to analyze on an HPLC column. From the above-described mixture, 10 µl was injected into an Agilent Zorbax C-18 reverse phase HPLC column (4.6 mm I.D., 250-mm length and 5-μm particle size) at 30 °C, equipped with a 205 nm UV detector. Acetonitrile (solvent A) and 30 mM phosphoric acid in water (solvent B) was used as mobile phase with a flow rate of 1 ml/min. The gradient started at 50% A followed by a linear increase to 90% A in 15 min. 100% A was then held for 10 min followed by a return to the initial condition in next 10 min. Under similar conditions, ricinoleic acid of different concentrations were run to prepare a standard curve to calculate the process yield (Additional file 6A).

### Structural Modeling of TON-LPL and rc-TGL

The structural models of TON-LPL and rc-TGL were obtained by I-TASSER on-line web server [37, 38]. The model quality was estimated by C-score and RMSD, as reported in the output of I-TASSER server. C-score is a confidence score for estimating the quality of predicted models by I-TASSER. C-score is typically in the range of − 5 to 2, where a C-score of higher value signifies a model with a high confidence and vice versa. The structures were visualized by discovery studio software suite of accelrys. These structural information were used to define the boundary of N-terminal replacement.

## Additional files

**Additional file 1.** Amino acid sequence of TON-LPL, rc-TGL and TLIP.

**Additional file 2.** SDS-PAGE of TON-LPL in whole cell lysate and cell free extract.

**Additional file 3: 3.1.** Gene designing strategy of rc-TGL. **3.2.** PCR and cloning of rc-TGL into pET23a expression vector. **3.3.** Primer sequences used to construct rc-TGL.

**Additional file 4.** Calibration curve of superdex-200 increase column.

**Additional file 5: A.** SDS-PAGE analysis of enzyme immobilization. **B.** Table showing enzyme activity in immobilization.

**Additional file 6.** HPLC based quantitative estimation of ricinoleic acid.

## Abbreviations

ABHDB: alpha/beta-hydrolase fold 3DM database
TON-LPL: *Thermococcus onnurineus* lysophospholipase
rc-TGL: recombinant triglyceride lipase
TLIP: *Thermomyces lanuginosus* lipase
PDB: protein data bank
RMSD: root mean square deviation
I-TASSER: iterative threading ASSEmbly refinement)
SOE-PCR: splicing overlap extension polymerase chain reaction
pNP: para-nitrophenol
AIM: autoinduction medium
IPTG: isopropyl β-d-1-thiogalactopyranoside

## References

[1] Anobom CD, Pinheiro AS, De-Andrade RA, Aguieiras ECG, Andrade GC, Moura MV, Rodrigo VA, Denise MF (2014). From structure to catalysis: recent developments in the biotechnological applications of lipases. Biomed Res Int.

[2] Hasan F, Shah AA, Hameed A (2006). Industrial applications of microbial lipases. Enzyme Microb Technol.

[3] Houde A, Kademi A, Leblanc D (2004). Lipases and their industrial applications: an overview. Appl Biochem Biotechnol.

[4] Jaeger KE, Schneidinger B, Rosenau F, Werner M, Lang D, Dijkstra BW, Klaus S, Albin Z, Manfred TR (1997). Bacterial lipases for biotechnological applications. J Mol Catal B Enzym.

[5] Schmid RD, Verger RL (1998). Interfacial enzymes with attractive applications. Angew Chem Int Ed Engl.

[6] Kumar A, Dhar K, Kanwar SS, Arora PK (2016). Lipase catalysis in organic solvents: advantages and applications. Biol Proced Online..

[7] Ramnath L, Sithole B, Govinden R (2017). Classification of lipolytic enzymes and their biotechnological applications in the pulping Industry. Can J Microbiol.

[8] Arpigny JL, Jaeger K (1999). Bacterial lipolytic enzymes: classification and properties. Biochem J..

[9] Anderson EM, Larsson KM, Kirk O (1998). One biocatalyst- many applications: the use of *Candida antarctica* B-lipase in organic synthesis. Biocatal. Biotransformation..

[10] Choudhury P, Bhunia B (2017). Industrial application of lipase: a review. Biopharm journal..

[11] Angajala G, Pavan P, Subashini R (2016). Lipases: an overview of its current challenges and prospectives in the revolution of biocatalysis. Biocatal Agric Biotechnol.

[12] Palomo JM, Muoz G, Fernández-Lorente G, Mateo C, Fernández-Lafuente R, Guisán JM (2002). Interfacial adsorption of lipases on very hydrophobic support (octadecyl-Sepabeads): immobilization, hyperactivation and stabilization of the open form of lipases. J Mol Catal B Enzym.

[13] Lenfant N, Hotelier T, Bourne Y, Marchot P, Chatonnet A (2013). Proteins with an alpha/beta hydrolase fold: relationships between subfamilies in an ever-growing superfamily. Chem Biol Interact.

[14] Dimitriou PS, Denesyuk A, Takahashi S, Yamashita S, Johnson MS, Nakayama T, Konstantin D (2017). Alpha/beta-hydrolases: a unique structural motif coordinates catalytic acid residue in 40 protein fold families. Proteins..

[15] Suplatov DA, Besenmatter W, Švedas VK, Svendsen A (2012). Bioinformatic analysis of alpha/beta-hydrolase fold enzymes reveals subfamily-specific positions responsible for discrimination of amidase and lipase activities. Protein Eng Des Sel.

[16] Kourist R, Jochens H, Bartsch S, Kuipers R, Padhi SK, Gall M, Bornscheuer UT (2010). The α/β-hydrolase fold 3DM database (ABHDB) as a tool for protein engineering. ChemBioChem.

[17] Secundo F, Carrea G, Tarabiono C, Gatti-Lafranconi P, Brocca S, Lotti M (2006). The lid is a structural and functional determinant of lipase activity and selectivity. J Mol Catal B Enzym.

[18] Skjold-Jørgensen J, Vind J, Moroz OV, Blagova E, Bhatia VK, Svendsen A, Keith SW, Morten JB (2017). Controlled lid-opening in *Thermomyces lanuginosus* lipase—an engineered switch for studying lipase function. Biochim Biophys Acta Proteins Proteom..

[19] Khan FI, Lan D, Durrani R, Huan W, Zhao Z, Wang Y (2017). The lid domain in lipases: structural and functional determinant of enzymatic properties. Front Bioeng Biotechnol..

[20] Yu XW, Zhu SS, Xiao R, Xu Y (2014). Conversion of a *Rhizopus chinensis* lipase into an esterase by lid swapping. J Lipid Res.

[21] Tang T, Yuan C, Hwang HT, Zhao X, Ramkrishna D, Liu D, Varma A (2015). Engineering surface hydrophobicity improves activity of *Bacillus thermocatenulatus* lipase 2 enzyme. Biotechnol J.

[22] Ikeda M, Clark DS (1998). Molecular cloning of extremely thermostable esterase gene from hyperthermophilic archaeon *Pyrococcus furiosus* in *Escherichia coli*. Biotechnol Bioeng.

[23] Alquéres SMC, Branco RV, Freire DMG, Alves TLM, Martins OB, Almeida RV (2011). Characterization of the recombinant thermostable lipase (Pf2001) from *Pyrococcus furiosus*: effects of thioredoxin fusion tag and triton X-100. Enzyme Res..

[24] Killens-Cade R, Turner R, Macinnes C, Grunden A (2014). Characterization of a thermostable, recombinant carboxylesterase from the hyperthermophilic Archaeon *Metallosphaera sedula DSM5348*. Adv Enzyme Res..

[25] Hotta Y, Ezaki S, Atomi H, Imanaka T (2002). Extremely stable and versatile carboxylesterase from a hyperthermophilic archaeon. Appl Environ Microbiol.

[26] Gao R, Feng Y, Ishikawa K, Ishida H, Ando S, Kosugi Y (2003). Cloning, purification and properties of a hyperthermophilic esterase from archaeon *Aeropyrum pernix* K1. J Mol Catal B Enzym.

[27] Park YJ, Choi SY, Lee HB (2006). A carboxylesterase from the thermoacidophilic archaeon *Sulfolobus solfataricus* P1; purification, characterization, and expression. Biochim Biophys Acta Gen Subj.

[28] Morana A, Di Prizito N, Aurilia V, Rossi M, Cannio R (2002). A carboxylesterase from the hyperthermophilic archaeon *Sulfolobus solfataricus*: cloning of the gene, characterization of the protein. Gene.

[29] Cui Z, Wang Y, Pham BP, Ping F, Pan H, Cheong GW, Zhang S (2012). High level expression and characterization of a thermostable lysophospholipase from *Thermococcus kodakarensis* KOD1. Extremophiles.

[30] Rusnak M, Nieveler J, Schmid RD, Petri R (2005). The putative lipase, AF1763, from *Archaeoglobus fulgidus* is a carboxylesterase with a very high pH optimum. Biotechnol Lett.

[31] Kim S, Lee W, Ryu Y (2008). Cloning and characterization of thermostable esterase from *Archaeoglobus fulgidus*. J Microbiol.

[32] Shao H, Xu L, Yan Y (2014). Biochemical characterization of a carboxylesterase from the archaeon *pyrobaculum* sp. 1860 and a rational explanation of its substrate specificity and thermostability. Int J Mol Sci.

[33] Zhang XY, Fan X, Qiu YJ, Li CY, Xing S, Zheng YT (2014). Newly Jian-He Xu identified thermostable esterase from *Sulfobacillus acidophilu*s: properties and performance in phthalate ester degradation. Appl Environ Microbiol.

[34] Chen CKM, Lee GC, Ko TP, Guo RT, Huang LM, Liu HJ, Yi-Fang H, Jei-Fu S, Andrew H, Wang J (2009). Structure of the alkalohyperthermophilic *Archaeoglobus fulgidus* lipase contains a unique C-terminal domain essential for long-chain substrate binding. J Mol Biol.

[35] Brzozowski AM, Savage H, Verma CS, Turkenburg JP, Lawson DM, Svendsen A, Patkar S (2000). Structural origins of the interfacial activation in *Thermomyces* (*Humicola*) *lanuginosa* lipase. Biochemistry.

[36] Cajal Y, Svendsen A, Girona V, Patkar SA, Alsina MA (2000). Interfacial control of lid opening in *Thermomyces lanuginosa* lipase. Biochemistry.

[37] Roy A, Kucukural A, Zhang Y (2010). I-TASSER: a unified platform for automated protein structure and function prediction. Nat Protoc.

[38] Zhang Y (2008). I-TASSER server for protein 3D structure prediction. BMC Bioinf..

[39] Rosano GL, Ceccarelli EA (2014). Recombinant protein expression in *Escherichia coli*: advances and challenges. Front Microbiol..

[40] Block H, Maertens B, Spriestersbach A, Brinker N, Kubicek J, Fabis R, Jörg L, Frank S (2009). Immobilized-metal affinity chromatography (IMAC): a review. Meth Enzymol..

[41] Vieille C, Zeikus GJ (2001). Hyperthermophilic enzymes: sources, uses, and molecular mechanisms for thermostability. Microbiol Mol Biol Rev.

[42] Fraser NJ, Liu JW, Mabbitt PD, Correy GJ, Coppin CW, Lethier M, Perugini MA, Murphy MJ, John GO, Martin W, Colin JJ (2016). Evolution of protein quaternary structure in response to selective pressure for increased thermostability. J Mol Biol.

[43] Tanakai Y, Tsumoto K, Yasutake Y, Umetsu M, Yao M, Fukada H, Isao T, Izumi K (2004). How oligomerization contributes to the thermostability of an archaeon protein: protein l-isoaspartyl-*O*-methyltransferase from *Sulfolobus tokodaii*. J Biol Chem.

[44] Kado Y, Inoue T, Ishikawa K (2011). Structure of hyperthermophilic β-glucosidase from *Pyrococcus furiosus*. Acta Crystallogr F Struct Biol Commun..

[45] Tokuriki N, Stricher F, Serrano L, Tawfik DS (2008). How protein stability and new functions trade off. PLoS Comput Biol.

[46] Siddiqui KS (2017). Defying the activity–stability trade-off in enzymes: taking advantage of entropy to enhance activity and thermostability. Crit Rev Biotechnol.

[47] Bloom JD, Wilke CO, Arnold FH, Adami C (2004). Stability and the evolvability of function in a model protein. Biophys J.

[48] Ragauskas AJ, Williams CK, Davison BH, Britovsek G, Cairney J, Eckert CA, William JF, Jason PH, David JL, Charles LL, Jonathan RM, Richard M, Richard T, Timothy T (2006). The path forward for biofuels and biomaterials. Science.

[49] Bateni H, Karimi K, Zamani A, Benakashani F (2014). Castor plant for biodiesel, biogas, and ethanol production with a biorefinery processing perspective. Appl Energy.

