# Supplementary material for: N-terminal domain replacement changes an archaeal monoacylglycerol lipase into a triacylglycerol lipase
Source: Biotechnol Biofuels. 2019 May 6;12:110. doi: 10.1186/s13068-019-1452-5 (PMC6501381; doi:10.1186/s13068-019-1452-5)
Supplement: Supplementary file 1 — Additional file 1. Amino acid sequence of TON-LPL, rc-TGL and TLIP. [file 13068_2019_1452_MOESM1_ESM.docx]

**Additional file 1**

**Amino acid sequence of TON-LPL, rc-TGL and TLIP**

**
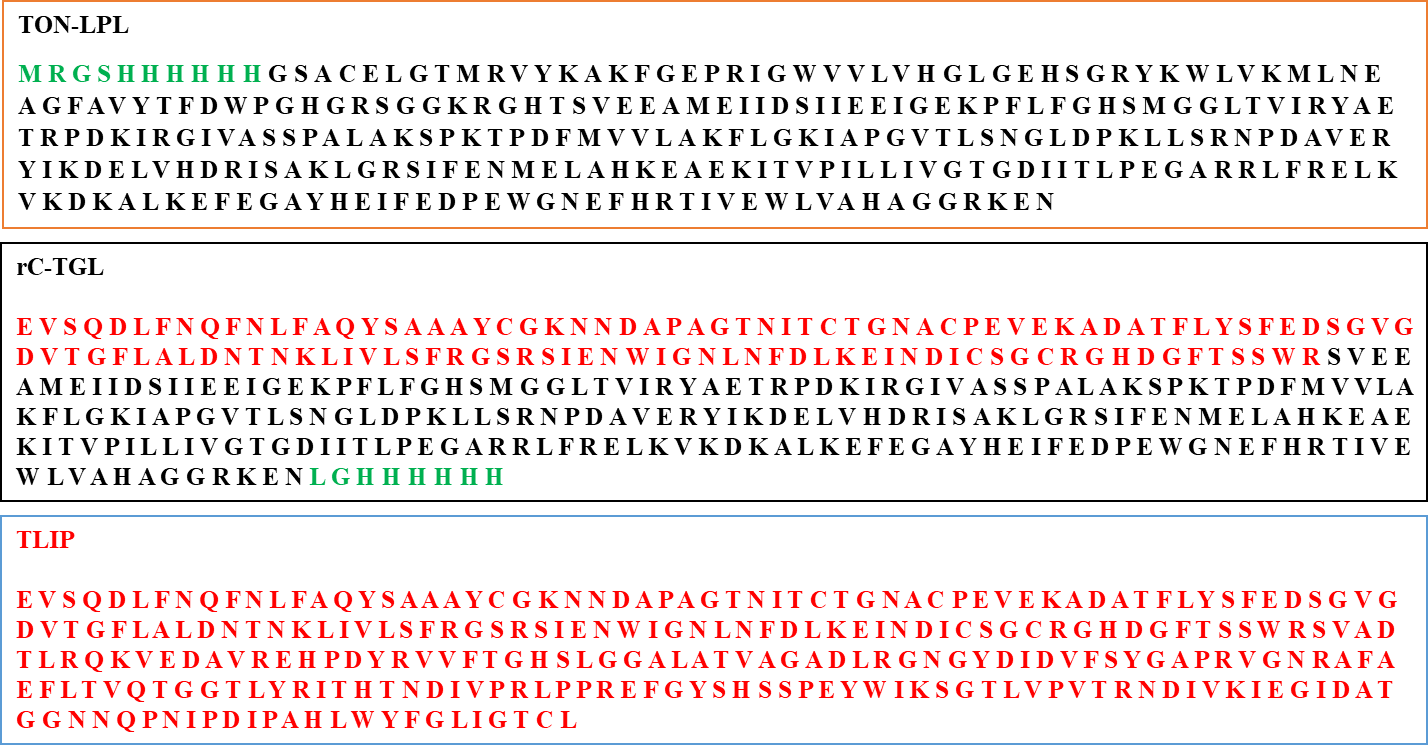
**

Additional file 1: The amino acid sequence of TON-LPL (black), TLIP (red) and rc-TGL (red /black) with the 6X-His tag (green) are shown.
