# Supplementary material for: N-terminal domain replacement changes an archaeal monoacylglycerol lipase into a triacylglycerol lipase
Source: Biotechnol Biofuels. 2019 May 6;12:110. doi: 10.1186/s13068-019-1452-5 (PMC6501381; doi:10.1186/s13068-019-1452-5)
Supplement: Supplementary file 2 — Additional file 2. SDS-PAGE of TON-LPL in whole cell lysate and cell free extract. [file 13068_2019_1452_MOESM2_ESM.docx]

**Additional file 2**

**SDS-PAGE of TON-LPL in whole cell lysate and cell free extract**

**
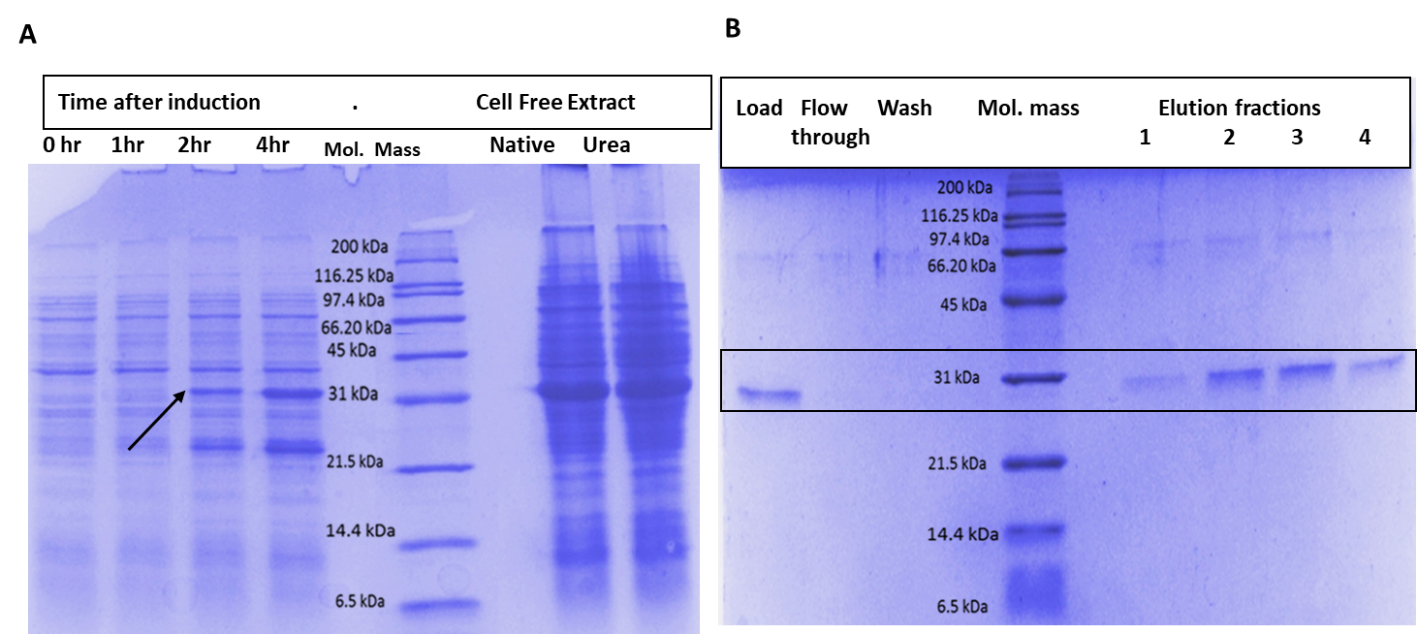
**

Additional file 2: (A) XL1 Cells were grown in LB medium and induced by 1mM IPTG at mid-exponential phase. At different time-intervals cell pellets were lysed into 5X-SDS-PAGE loading buffer and samples were analyzed by SDS-PAGE. The induced TON-LPL is marked by black arrow. In the right side of the Figure 2A, the cell pellets were lysed into native (without urea) and urea lysis buffer and the cell free extract were analyzed by SDS-PAGE. The corresponding induced band was present in both cell free extract (marked by black arrow).

(B) TON-LPL was loaded in urea containing buffer onto Ni-NTA affinity column and the bound proteins were eluted in urea free buffer to obtain the refolded TON-LPL. Subsequently, the refolded TON-LPL eluted after on-column refolding was reloaded on Ni-NTA affinity column. Here, the refolded TON-LPL binds to column and elutes in presence of imidazole buffer unlike the induced protein (TON-LPL) in the cell free extract. Lanes are marked for different steps of Ni-NTA affinity column chromatography. Load represents the refolded TON-LPL.
