# Supplementary material for: N-terminal domain replacement changes an archaeal monoacylglycerol lipase into a triacylglycerol lipase
Source: Biotechnol Biofuels. 2019 May 6;12:110. doi: 10.1186/s13068-019-1452-5 (PMC6501381; doi:10.1186/s13068-019-1452-5)

**Additional file 3**

**Additional file 3.1: Gene designing strategy of rc-TGL**

**328**

**118**

TON-LPL of an archaeon *Thermococcus onnurineus*

*(strain NA1)*

**1**

Lid subdomain from *Thermomyces lanuginosus* (TLIP)

**C**

**N**

Fragment 2

Fragment 1

Additional file 3.1: Lid engineering approach to create a novel enzyme where TON-LPL is parent and lid subdomain (shown in grey) donor is a well-studied lipase *Thermomyces lanuginosus* (TLIP). The position 1 to 118 represents the amino acids from TLIP sequence and 119-328 represent the amino acids from TON-LPL.

**Additional File 3.2: PCR and cloning of rc-TGL into pET23a expression vector**


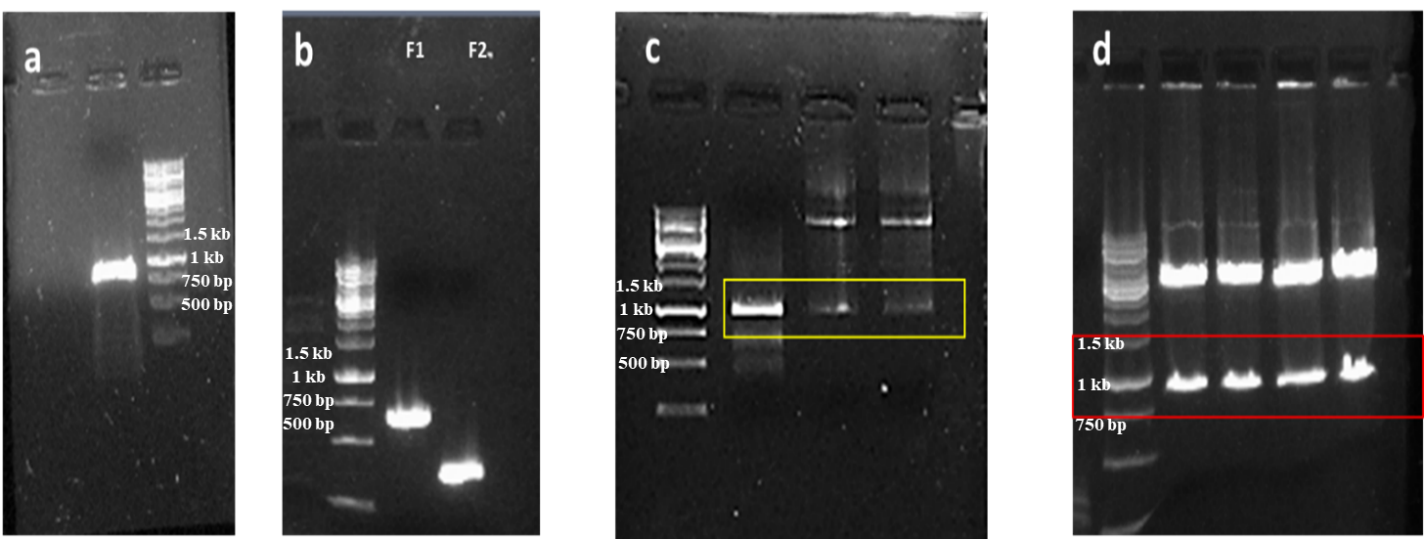


Additional file 3.2 : Agarose gel electrophoresis of (a) PCR amplified product of TON-LPL; (b) PCR amplified fragments (F1 and F2) for construction of rc-TGL; (c) Complete rc-TGL gene generated by spliced overlap extension PCR (SOE-PCR); and (d) Restriction digestion of selected positive colonies to confirm cloned rc-TGL in pET23a expression vector. The marker used for reference is of Thermo Scientific (Gene Ruler) 1kb DNA ladder.

**Additional file 3.3: Primer sequences used to construct rc-TGL**


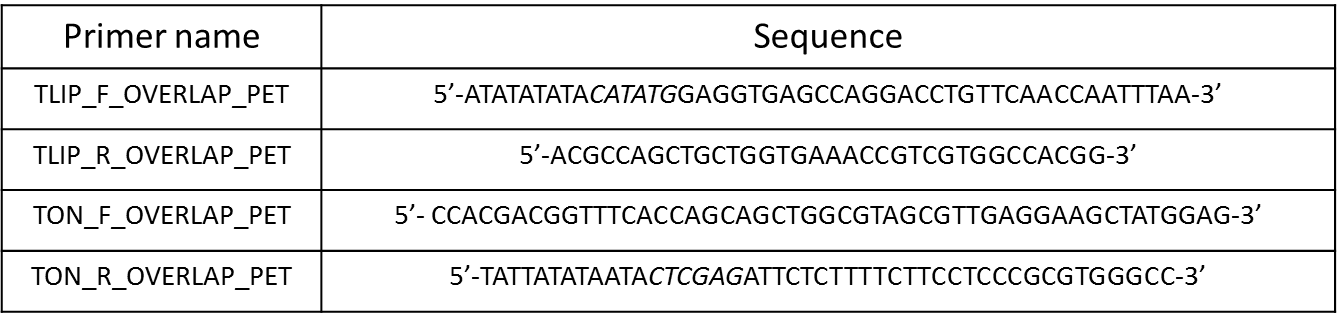

Supplement: Supplementary file 3 — Additional file 3: 3.1. Gene designing strategy of rc-TGL. 3.2. PCR and cloning of rc-TGL into pET23a expression vector. 3.3. Primer sequences used to construct rc-TGL. [file 13068_2019_1452_MOESM3_ESM.docx]
