# Supplementary material for: N-terminal domain replacement changes an archaeal monoacylglycerol lipase into a triacylglycerol lipase
Source: Biotechnol Biofuels. 2019 May 6;12:110. doi: 10.1186/s13068-019-1452-5 (PMC6501381; doi:10.1186/s13068-019-1452-5)
Supplement: Supplementary file 4 — Additional file 4. Calibration curve of superdex-200 increase column. [file 13068_2019_1452_MOESM4_ESM.docx]

**Additional file 04**

**Calibration curve of superdex-200 increase column**


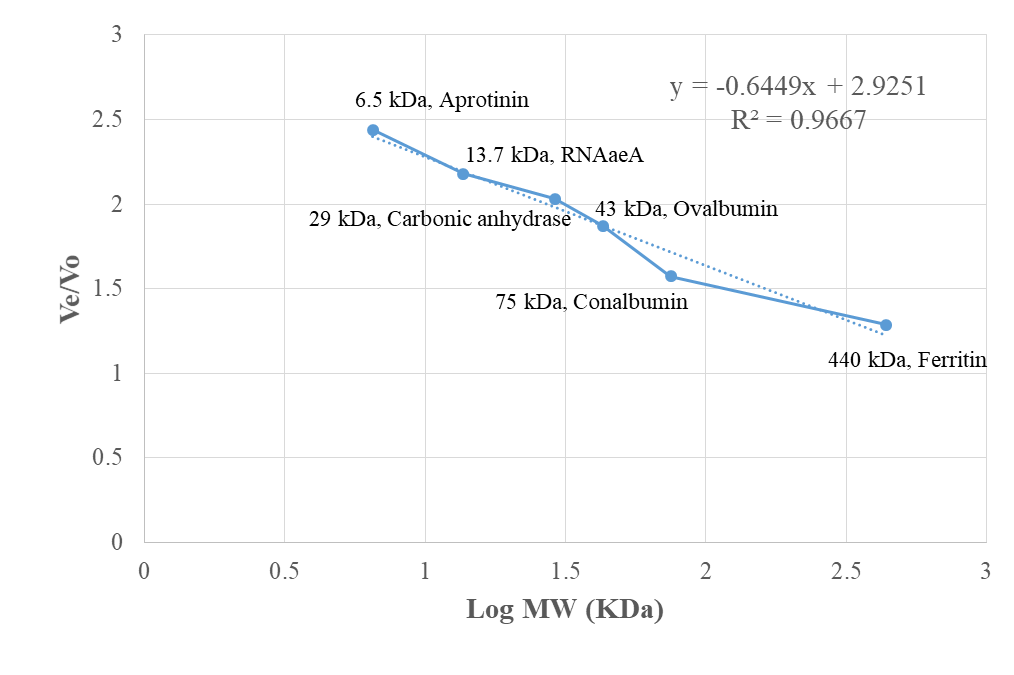


Additional file 4: Standard calibration curve of superdex-200 increase column to estimate the molecular weights. Here Ve and Vo corresponds to elution volume and void volume of the column respectively.
