# Supplementary material for: N-terminal domain replacement changes an archaeal monoacylglycerol lipase into a triacylglycerol lipase
Source: Biotechnol Biofuels. 2019 May 6;12:110. doi: 10.1186/s13068-019-1452-5 (PMC6501381; doi:10.1186/s13068-019-1452-5)

**Additional file 5: SDS-PAGE analysis of enzyme immobilization**


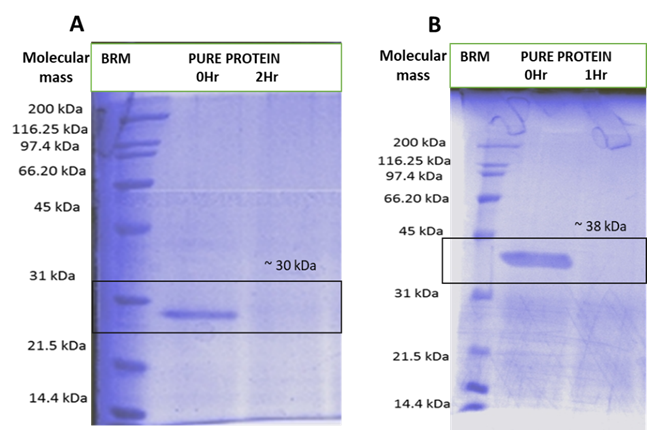


Additional file 5A: SDS-PAGE of immobilization profile of protein TON-LPL (A) and rc-TGL (B) on Indion PA500 resin respectively with protein marker on left and incubation time of each lane is labeled on top. At each time the supernatant was loaded on SDS-PAGE.

**Additional file 5B: Table showing enzyme activity in immobilization**


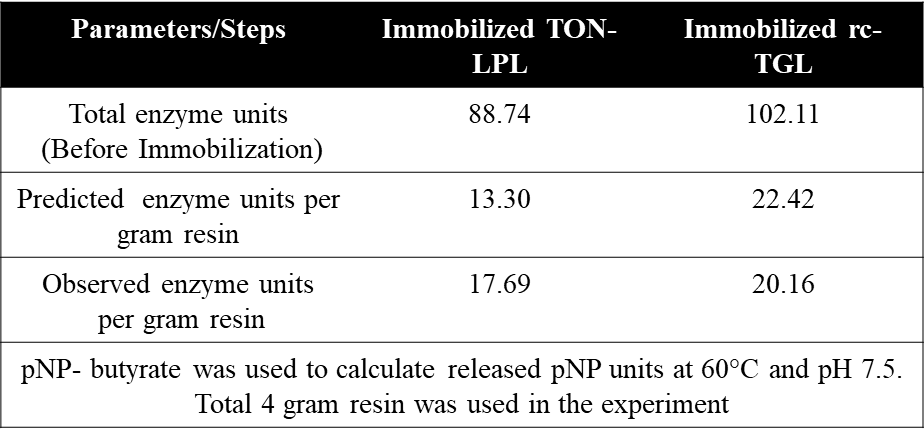

Supplement: Supplementary file 5 — Additional file 5: A. SDS-PAGE analysis of enzyme immobilization. B. Table showing enzyme activity in immobilization. [file 13068_2019_1452_MOESM5_ESM.docx]
