# Supplementary material for: N-terminal domain replacement changes an archaeal monoacylglycerol lipase into a triacylglycerol lipase
Source: Biotechnol Biofuels. 2019 May 6;12:110. doi: 10.1186/s13068-019-1452-5 (PMC6501381; doi:10.1186/s13068-019-1452-5)
Supplement: Supplementary file 6 — Additional file 6. HPLC based quantitative estimation of ricinoleic acid. [file 13068_2019_1452_MOESM6_ESM.docx]

**Additional file 6: HPLC based quantitative estimation of ricinoleic acid**

**A**

**
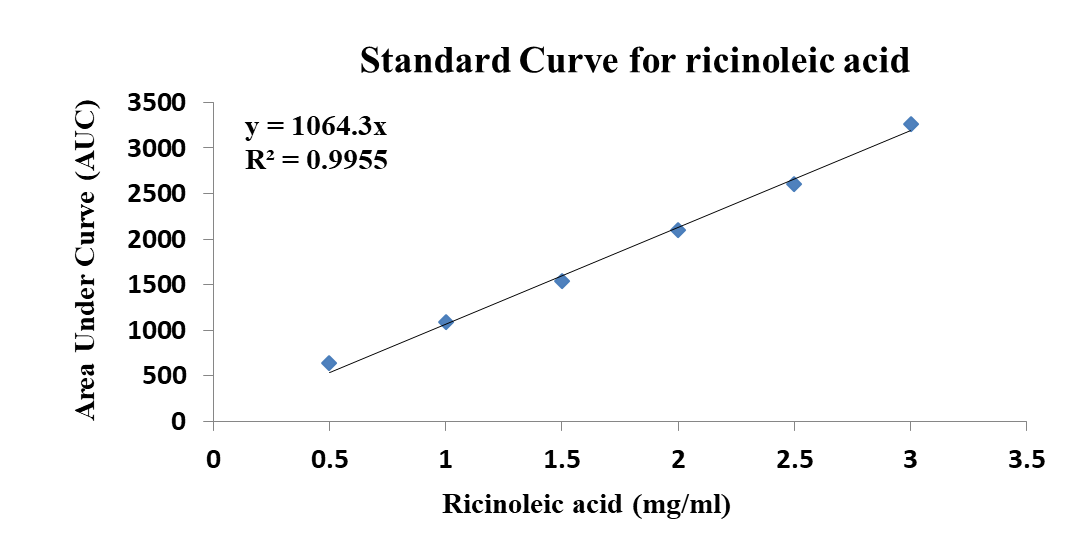
**

**B**

**TLIP (Lab)**

**TLIP**

**(Commercial)**

**rC-TGL**

**TON-LPL**

**B**

**Ricinoleic acid**

Additional file 6B: HPLC profile on Agilent Zorbax C-18 reverse phase HPLC column is shown of castor oil hydrolysis by TLIP (red, black), rc-TGL (blue) and TON-LPL (green). The assays were performed overnight at 55°C. The product formed after castor oil hydrolysis at 13.5 minutes is Ricinoleic acid.
